# Molecular basis of the attenuated phenotype of human APOBEC3B DNA mutator enzyme

**DOI:** 10.1093/nar/gkv935

**Published:** 2015-09-17

**Authors:** Vincent Caval, Mohamed S. Bouzidi, Rodolphe Suspène, Hélène Laude, Marie-Charlotte Dumargne, Anu Bashamboo, Thomas Krey, Jean-Pierre Vartanian, Simon Wain-Hobson

**Affiliations:** 1 Molecular Retrovirology Unit, Institut Pasteur, 28 rue du Dr. Roux, 75724 Paris cedex 15, France; 2 Human Developmental Genetics Unit, Institut Pasteur, 28 rue du Dr. Roux, 75724 Paris cedex 15, France; 3 Structural Virology Unit, Institut Pasteur, 28 rue du Dr. Roux, 75724 Paris cedex 15, France

## Abstract

The human APOBEC3A and APOBEC3B genes (A3A and A3B) encode DNA mutator enzymes that deaminate cytidine and 5-methylcytidine residues in single-stranded DNA (ssDNA). They are important sources of mutations in many cancer genomes which show a preponderance of CG->TA transitions. Although both enzymes can hypermutate chromosomal DNA in an experimental setting, only A3A can induce double strand DNA breaks, even though the catalytic domains of A3B and A3A differ by only 9% at the protein level. Accordingly we sought the molecular basis underlying A3B attenuation through the generation of A3A-A3B chimeras and mutants. It transpires that the N-terminal domain facilitates A3B activity while a handful of substitutions in the catalytic C-terminal domain impacting ssDNA binding serve to attenuate A3B compared to A3A. Interestingly, functional attenuation is also observed for the rhesus monkey rhA3B enzyme compared to rhA3A indicating that this genotoxic dichotomy has been selected for and maintained for some 38 million years. Expression of all human ssDNA cytidine deaminase genes is absent in mature sperm indicating they contribute to somatic mutation and cancer but not human diversity.

## INTRODUCTION

Mutation-selection drives the oncogenic process (1,2). While replication errors and exogenous sources of DNA damage such as UV light, mutagens and free radicals have long been appreciated to be sources of somatic diversity driving oncogenesis (3), the contribution of two endogenous DNA mutator enzymes has only recently come to the fore (2,4–9). The APOBEC3A (A3A) and APOBEC3B (A3B) enzymes are cytidine deaminases, converting cytidine residues into uridine bases in single stranded DNA (ssDNA) (6,9–11). The non-canonical uracil bases are removed by uracil N-glycosylase (UNG) generating abasic sites in ssDNA, which in turn are cleaved by apurinic/apyrinidic endonucleases, APE1 and APE2. Together, the three enzymes effectively function as a cytidine endonuclease, promoting apoptosis or DNA repair depending on the extent of DNA damage.

Although the A3 enzymes were initially described as innate immune restriction factors for many DNA viruses, retroviruses and retroelements, A3A and A3B have emerged as sources of the large number of CG→TA somatic mutations in cancer genomes and nuclear DNA (nuDNA) in experimental settings (2,4–9). A3 editing leaves a tell tale 5′TpC mutational signature, where C is the edited base, which shows up in many cancer genomes (2,4,8,12,13). Epidemiological data links A3A to the development of breast, ovarian and hepatitis B virus associated liver cancer (7,14–16). While both A3A and A3B can deaminate 5-methylcytidine residues (6,17), the two enzymes are not orthologous for A3B overexpression fails to produce detectable double stranded DNA breaks (DSBs), unlike A3A (6).

A3A is a 26 kDa enzyme with a single zinc binding domain (18). By contrast A3B is a 46 kDa enzyme made up of two zinc binding domains, the inactive N-terminal domain (A3Bn) domain (19–21) being more closely related to the N-terminal domains of APOBEC3DE, F and G, while the catalytically active C-terminal domain, A3Bc, arose from duplication of the *A3A* gene (22–24). Because of this, A3A and A3Bc are 91% identical at the protein level, starting from A3Ap2 initiating methionine (6). To explore the molecular basis of A3B attenuated DSB phenotype compared to A3A, a large number of chimeras and mutants were generated and their genotoxicity assessed. The N-terminal domain of A3B and at least three residues involved in DNA binding by the A3Bc catalytic domain contribute to A3B attenuation. The rhesus monkey A3B enzyme is equally attenuated compared to rhA3A indicating that the trade off between A3A and A3B has been maintained for at least 38 million years.

## MATERIALS AND METHODS

### Plasmids

APOBEC3A, APOBEC3Ap2 and APOBEC3B expression plasmids and catalytic mutants have been previously described (6,9,25) and were used to generate chimeras by PCR (Supplementary Table S1) or site directed mutagenesis (GeneArt® Site-Directed Mutagenesis System, Life Technologies) (Supplementary Table S2). To overcome toxicity in *E. coli*, A3BnA and rhesus APOBEC3B (RhA3B) plasmids were synthetized retaining intron 7, and subsequently cloned into pcDNA3.1D/V5-His-TOPO vector (Life Technologies). Rhesus-A3A expression plasmid was previously described and used to generate the rhesus-A3Ap2 plasmid using PCR. All constructs were grown in *E. coli* TOP10 cells (Life Technologies) and verified by sequencing.

### Cells

Quail QT6 fibroblast cells were maintained in HAM's F40 medium (Eurobio), supplemented with 1% chicken serum, 10% FCS, 5% tryptose phosphate, 2 mM l-glutamine, 50 U/ml penicillin and 50 mg/ml streptomycin. Human HeLa cells and 293T-UGI cells stably expressing *Bacillus subtilis* phage uracil-DNA glycosylase inhibitor (UGI) were maintained in DMEM glutamax medium (Life Technologies) supplemented with 10% FCS, 50 U/ml penicillin and 50 mg/ml streptomycin.

### Transfections

One million QT6 cells were co-transfected with 0.5 μg of pCayw HBV coding plasmid and 1.5 μg APOBEC3 expression plasmids using JetPrime (Polyplus) following manufacturer's recommendations and harvested 48 hours post-transfection. For single plasmid transfections, 8 × 10^5^ of HeLa, 293T-UGI, cells were transfected using 2 μg APOBEC3 expression plasmids using JetPrime (Polyplus) following manufacturer's recommendations and harvested 48 hours post-transfection. For immunofluorescence labeling, 5 × 10^4^ HeLa cells grown on chamber slides (LabTek) were transfected with either 1 μg APOBEC3 expression plasmids using Fugene HD (Roche) following manufacturer's recommendations.

### Western blotting

Transfected cells were resuspended in lysis buffer (0.5% Nonidet P-40, 20 mM Tris–HCl pH7.4, 120 mM NaCl, and 1 mM EDTA) supplemented with Complete Protease Inhibitor Mixture (Roche Applied Science). Cell lysates were incubated on ice for 20 min and then clarified by centrifugation at 14 000 × g for 30 min. Western blot analysis on cell lystates was carried out according to standard procedures. After blocking, membranes were probed with 1/5000 diluted mouse monoclonal antibody specific for the V5 epitope (Life Technologies) in PSB–0.1% Tween 5% dry milk applied overnight. After PBS–Tween washings and incubation with an anti-mouse IgG horseradish peroxidase-coupled secondary antibody (Amersham), the membrane was revealed by enhanced chemiluminescence (Pierce). β-Actin was used as a loading control using 1/2000 diluted mouse monoclonal antibody specific for β-actin (Sigma).

### Immunofluorescence

After PBS washings, transfected HeLa cells grown on chamber slides were fixed with 4% PFA for 15 min. After PBS washing cells were incubated in 50/50 acetone/methanol for 20 min. Mouse monoclonal anti-V5 antibody (Life Technologies), and rabbit monoclonal anti-FLAG (Sigma–Aldrich) were incubated at 1/200 for 1 h at room temperature, followed by incubation with a mouse specific Alexa-488 goat antibody and rabbit specific alexa-555 conjugated donkey antibody 1 h at room temperature in the dark. After washing, slides were mounted with Vectashield imaging medium containing DAPI (Vector Laboratories). Imaging was performed using Leica SP5 confocal microscope.

### *In vitro* deamination assay

At 72 h after transfection, APOBEC3 transfected 293T cells were extensively wash with PBS and mechanically harvested. Total proteins were extracted using specific lysis buffer (25 mM HEPES pH7.4, 10% glycerol, 150 mM NaCl, 0.5% Triton X-100, 1 mM EDTA, 1 mM MgCl_2_, 1 mM ZnCl_2_) supplemented with protease inhibitors, and submitted to sonication. Deaminase activity was assessed by incubating whole cell lysates with 1 pmol DNA oligonucleotide 5′-(6-FAM)-AAATTCTAATAGATAATGTGA-(TAMRA)-3′ in presence of 0.4 unit UDG (NEB) in a 20 mM Tris–HCl, 1 mM DTT, 1 mM EDTA reaction buffer. After 2 h incubation at 37°C, generated abasic sites were cleaved by heating 2 min at 95°C, and endpoint fluorescence were measured using realplex^2^ Mastercycler (BioRad) with FAM setting and background fluorescence obtained with mock-transfected cells set as negative control. Results are normalized to the quantity of protein using Pierce BCA Protein Assay Kit (Thermo Scientific).

### DNA extraction and 3DPCR amplification

Total DNA from transfected cells was extracted using the MasterPure™ complete DNA and RNA purification kit (Epicentre) and resuspended in 30 μl sterile water. All amplifications were performed using first-round standard PCR followed by nested 3DPCR (9,26). PCR was performed with 1 U Taq DNA polymerase (Eurobio) per reaction.

### FACS analysis of double strand breaks

At 48 h after transfection, cells were washed with PBS, fixed in 2–4% ice-cold paraformaldehyde (Electron Microscopy Sciences) for 10 min and permeabilized in 90% ice-cold methanol (Sigma) for 30 min. After washing with PBS, cells were incubated with 1:200 diluted mouse anti-V5 antibody (Life Technologies) in PBS-BSA 0.5% for 1 h. After PBS washings incubation with 1:500 diluted Alexa Fluor 633 F(ab’)_2_ fragment of goat anti-mouse IgG (H+L) (Life Technologies) was performed for 45 min. DNA double strand breaks were analyzed by staining for 1 h with 1:50 diluted Alexa Fluor 488-conjugated rabbit monoclonal anti-γH2AX (20E3) antibody (Cell Signaling). All incubation steps were performed on ice. Stained samples were analyzed on a FACSCalibur using CellQuest Pro (BD Biosciences, version 5.2) and data were analyzed with FlowJo software (Tree Star Inc. version 8.7.1).

### Sperm RNAseq

Spermatozoa from three individuals were selected and separated from somatic cells by centrifuging 1–2 ml of semen through a two-layer (45% and 90%) gradient of Percoll at 400 × g for 20 min. The fraction in the 90% layer was washed in 2 ml of Tyrode (Eurobio) and centrifuged at 400 × g for 10 min. The pellet was collected with care to avoid somatic cell contamination. The pellet was washed twice in 1× PBS and RNA was isolated using RNeasy^®^ mini kit (Qiagen). Genomic DNA contamination was eliminated first by performing on-column DNase Digestion with the RNase-free DNase Set (Qiagen). Subsequently, the purified RNA samples were subjected to RNase-free DNase treatment (TURBO™ DNase, Ambion Inc.).

RNAseq analysis was performed using SOLiD™ Whole Transcriptome analysis kit for whole transcriptome libraries (Applied biosystems™) following the manufacturer's instruction. Briefly, total RNA isolated from spermatozoa was fragmented, purified, linked to adaptors and reverse transcribed to cDNA. This double stranded template was size selected on TBE-urea gel and DNA fragments between 100 and 200 bp in length were used to construct a SOLiD™ whole transcriptome library (Applied Biosystems SOLiD™ 3 System Templated Bead Preparation Guide) that was subsequently sequenced.

gColor Space sequence reads were mapped and quantified using the ABI Bioscope 1.3 Whole Transcriptome Analysis pipeline, the Integromics SeqSolve analysis software and a set of *ad hoc* perl scripts. The reference sequence datasets considered were the UCSC hg19 repeat-masked genome sequence and the NCBI RefSeq transcriptome dataset (September 2010). A purity score (calculated with a perl script) was assessed for each sample in order to evaluate the relative proportion of transcripts originating from cell types other than spermatozoa as described elsewhere (27). Purity was defined as the ratio of each of the potential ‘contaminants’ as a function of the average intensity for the sperm-specific *PRM1* and *PRM2* transcripts.

### Structural analysis

Figures were prepared with Pymol (http://www.pymol.org). The calculation of p*K*_a_ values of individual residues was performed using PROPKA 3.0 (28) on the first three models in their order of appearance in the coordinate file (PDB: 2M65). Further representations used the first model of the coordinate file. The electrostatic potential, represented in the figures ramp-colored from red (negative) to blue (positive) through white (neutral), was calculated using APBS (29) and contoured on a scale ranging from −5 to 5 kT/e. Mutagenesis was performed using Pymol choosing the most common rotamer for this residue that did not cause clashes.

## RESULTS

### Both N- and C-terminal A3B domains impact editing

**Figure 1.**
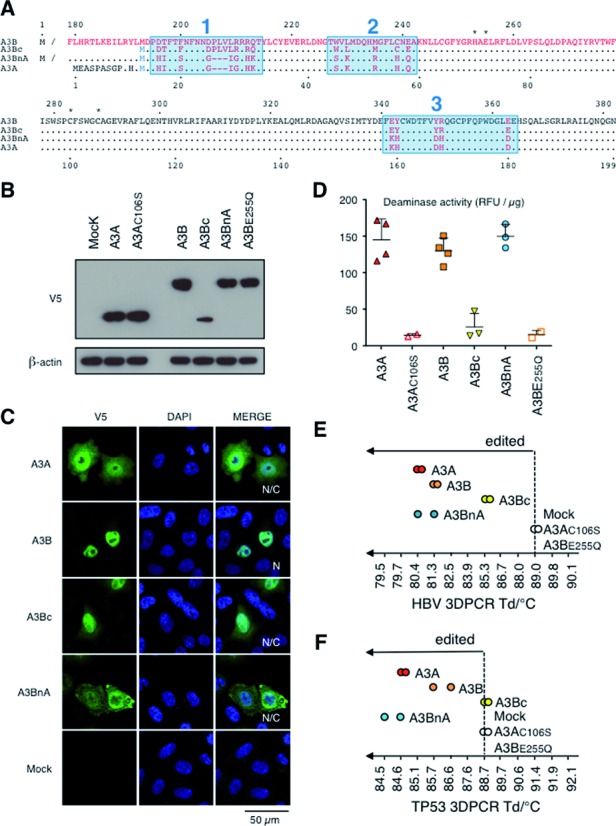
A3A/A3B chimeric constructions. (**A**) CLUSTALW alignment of A3A, A3B A3BnA, A3Bc. Mutational clusters are boxed in blue and residues involved in zinc coordination are represented by asterisks. Variable residues are depicted in red. Numbering above is that of A3B while that below is of A3A. (**B**) Western-blot analysis of V5-tagged APOBEC3 in human HEK 293T cells. β-Actin was used as loading control. (**C**) Cellular localization of A3 proteins. Confocal microscopy of V5 tagged A3 proteins performed in HeLa cells 24 h post-transfection. Nuclei are stained using DAPI. N: nuclear localization, N/C: nucleo-cytoplasmic localization. (**D**) *In vitro* deamination assay performed on TAMRA–FAM-coupled oligonucleotide using transfected 293T lysates from two experiments performed in duplicates. Background fluorescence obtained with mock-transfected cells was subtracted. RFU, Relative Fluorescence Unit. (**E**) 3D-PCR gel gradients for HBV DNA editing by A3 proteins. (**F**) TP53 specific 3DPCR gels after 293T-UGI transfections with A3 proteins.

In order to confer DSB formation, extensive mutagenesis of the catalytic C-terminal domain of A3B was first made. However, none of a series of 12 mutants encoding 1–3 residue substitutions as well as an A3B specific three-residue deletion (Supplementary Figure S1A) was able to rescue the DSB formation phenotype as assessed by FACS analysis of γH2AX staining, suggesting that A3B attenuation involved multiple residues. To attack the problem from a different angle, a chimeric construct was used whereby the A3Bc domain was replaced by A3A (A3BnA). A second construct comprising only A3Bc was made using the equivalent of the second Met^i^ in the A3A sequence, which leads to the functional A3AP2 isoform (Figure 1A) (25,30). A3BnA was well expressed while steady state A3Bc concentrations were lower than for A3A (Figure 1B). Interestingly, if A3Bc displayed a nucleo-cytoplasmic distribution similar to A3A, the A3BnA chimera was mostly found in the nucleus although retained some cytoplasmic distribution, suggesting that residues in both A3B domains are required for A3B localization, which is strictly nuclear (Figure 1C).

To evaluate deaminase activity, A3BnA and A3Bc-V5 tagged plasmids were transfected into 293T cells and cell lysates used in an *in vitro* FRET based assay where C to U deamination of a TAM-FAM labeled DNA oligonucleotide allows fluorescence detection after uracil-DNA glycolase (UNG) cleavage (6,11). While A3BnA displayed cytidine deaminase activity equal to A3A and A3B, only very weak activity was observed with A3Bc (Figure 1D). As HBV genome editing has proven to be a very sensitive assay for A3 activity *in vivo* (26), QT6 cells which do not show an endogenous editing background were transfected with A3B plasmids along with the pCayw HBV infectious molecular clone and HBV editing analyzed by 3DPCR. This technique allows recovery of AU rich A3-edited DNA at lower PCR denaturation temperatures compared to unedited DNA (31). For all constructs, HBV DNA was recovered at temperatures below the limiting denaturation temperature of *T*_d_ = 89.0°C corresponding to unedited DNA obtained with inactive A3BE255Q or A3AC106S mutants (Figure 1E, Supplementary Figure S2A). Editing was confirmed by cloning and sequencing of 3DPCR products. If A3BnA activity was on a par with A3A and A3B, A3Bc again proved to be less active (Figure 1E, Supplementary Figure S2A).

To assess the editing of nuclear DNA (nuDNA), 293T-UGI cells, stably expressing the *B. subtilis* UNG inhibitor (UGI) were transfected (6,9). Using 3DPCR specific for the cellular TP53 gene, edited nuDNA was recovered following transfection. As previously described, A3A consistently outperformed A3B with Tds down to 84.6°C compared to 85.7–86.6°C (6). A3BnA was able to hypermutate nuDNA, just like A3A and A3B while A3Bc did not (Figure 1F, Supplementary Figure S2B). As 3DPCR is not a quantitative technique, genotoxicity was quantitated using a more macroscopic indicator of A3 damage, γH2AX histone phosphorylation typical of DSB formation, was then used to assess A3-V5-tagged positive cells 48h post transfection.

**Figure 2.**
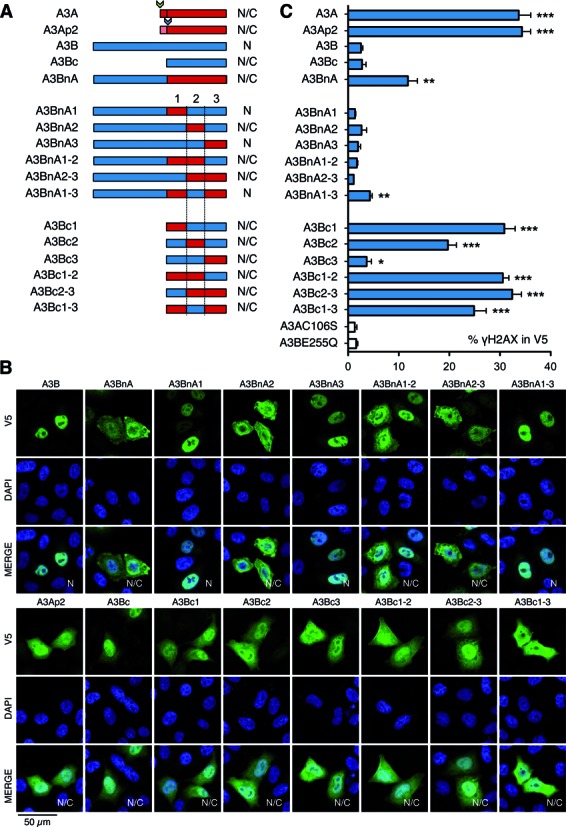
Schematic representation of A3BnA and A3Bc and cellular localization of A3 proteins. (**A**) Schematic representation of A3BnA and A3Bc derived chimeras. (**B**) Cellular localization of A3 proteins. Confocal microscopy of V5 tagged A3 proteins performed in HeLa cells 24 h post-transfection. Nuclei are stained using DAPI. N: nuclear localization, N/C: nucleo-cytoplasmic localization. (**C**) FACS analysis of γH2AX-positive HeLa cells gated on V5-positive cells after A3 48 h post-transfection. Error bars represent SD from six independent transfections. Differences compared with A3AC106S catalytic mutant were calculated using the Mann–Whitney test (*P < 0.1, **P < 0.01, ***P < 0.001).

Both A3B and A3Bc did not make detectable DSBs. By contrast A3BnA transfection resulted in significant levels of γH2AX phosphorylation albeit ∼50% compared to the A3Ap1 and A3Ap2 controls indicating that A3Bn can attenuate A3A activity (Figure 2C, Supplementary Figure S3A). Given these results, A3BnA mutants can be used for loss of function studies while A3Bc can be used to screen for gain of function mutants.

As all 18 residues (9%) distinguishing A3Ap2 from A3Bc were located in three regions referred to as mutation clusters 1, 2 and 3 (Figure 1A), six A3BnA and six A3Bc chimeras were made (Figure 2A). Expression levels were comparable (Supplementary Figure S1B) although there were subtle differences in nuclear localization, which was completely nuclear for A3BnA1, A3BnA3 and A3BnA1–3, suggesting that some A3B residues present in cluster 2 are necessary for complete nuclear addressing (Figure 2B). For the A3BnA derivatives exchange of almost any region of the C-terminal A3A domain resulted in loss of DSB formation (Figure 2C). By contrast, A3Bc mutation cluster 1 (A3Bc1) restored DSB formation to levels comparable to A3A and A3Ap2. The second cluster of mutations (A3Bc2) restored DSB formation but not quite as well, while A3Bc3 had a weak but significant effect (Figure 2C). Clusters 2 and 3 mutations combined were comparable to A3Bc1 and positive controls, A3Ap1 or A3Ap2.

### Molecular determinants of A3Bc attenuation

**Figure 3.**
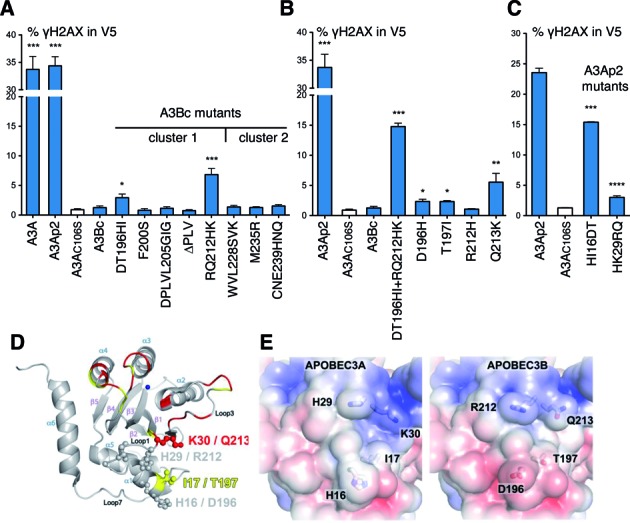
FACS analysis of γH2AX-positive HeLa cells and representation of the A3A structure. (**A**) FACS analysis of γH2AX-positive HeLa cells gated on V5-positive cells after A3B mutants at 48 h post-transfection. Error bars represent SD from six independent transfections. Differences compared with A3AC106S catalytic mutant were calculated using the Mann–Whitney test (*P < 0.1, **P < 0.01, ***P < 0.001). (**B**) FACS analysis for quadruple and single residue A3Bc mutants; annotated as in (A). (**C**) FACS analysis for A3Ap2 mutants at 48 h post-transfection; annotated as in (A). (**D**) Cartoon representation of the A3A structure. Side chains of residues identified by mutagenesis are shown as ball-and-sticks. Residues reported to undergo chemical shift upon nucleotide addition are colored in red (>0.05 ppm) and yellow (0.028–0.005 ppm). The zinc atom is represented as blue sphere and labeling refers to A3A/A3B numbering. (**E**) Molecular surface representation of the A3 enzymes, colored according to electrostatic potential (−5 kT/e [red] to 5 kT/e [blue]) calculated using APBS. Amino acid differences between A3 enzymes have an impact on the electrostatic surface potential.

To pinpoint gain of function mutations in A3Bc1 and A3Bc2 constructs conferring DSB formation, smaller combinations of A3Ap2 residues were introduced into the A3Bc coding plasmid (Figure 3A). Of all the constructs tested, only the RQ212HK, and to a lesser degree the DT196HI mutant, were found to induce significant DSB breaks (Figure 3A, Supplementary Figure S3B). The combined quadruple DT196HI+RQ212HK mutant recovered further DSB activity, more so than the sum of the two, but still only ∼50% DSB formation compared to A3Ap1 or A3Ap2 (Figure 3B, Supplementary Figure S3B), suggesting that other substitutions in mutant cluster 1 that do not score positive alone, when combined with DT196HI and RQ212HK contribute to genotoxicity. Single residue mutagenesis showed that the Q213K substitution was the most important with D196H and T197I scoring just above background levels (Figure 3B). Interestingly, reciprocal mutagenesis of A3Ap2 showed that the HK29RQ (A3A numbering, Figure 1A) construct attenuated DSB formation more than the HI16DT exchange mirroring the changes on the A3Bc background (Figure 3C, Supplementary Figure S3C). An NMR analysis of ssDNA binding to A3A identified K30 and to a lesser extent I17 residues (respectively Q213 and T197 on A3B, Figure 1A) as undergoing chemical shift changes, stressing their participation in substrate binding (32). These residues participate in binding the ssDNA backbone (Figure 3D and E).

### Conserved A3Bc attenuation

**Figure 4.**
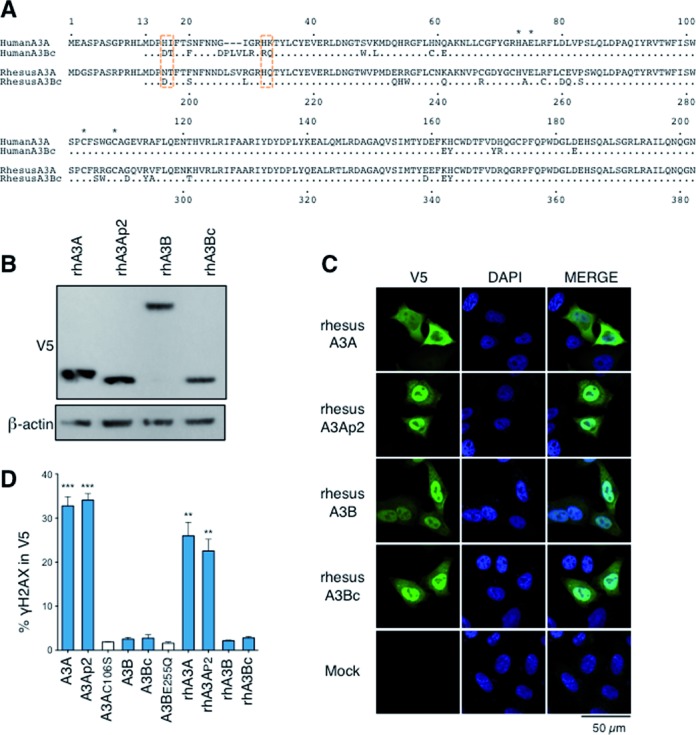
Alignment of A3A/A3Bc proteins and cellular localization of Rhesus-A3 proteins. (**A**) CLUSTALW alignment of A3A/A3Bc proteins from human and rhesus monkey. Critical functional residues distinguishing human A3A/A3Bc are boxed in orange while residues involved in zinc coordination are represented by an asterisk. Numbering above is that of human A3A while that below is of rhA3B. (**B**) Western-blot analysis of V5-tagged rhesus-A3 proteins in human HEK 293T cells. β-Actin was used as loading control. (**C**) Cellular localization of Rhesus-A3 proteins. Confocal microscopy of V5 tagged A3 proteins performed in HeLa cells 24 h post-transfection. Nuclei are stained using DAPI. (**D**) Fluorescence-activated cell sorting (FACS) analysis of γH2AX-positive HeLa cells gated on V5-positive cells after A3B mutants transfection at 48 h. Error bars represent SD from four independent transfections. Differences compared with A3AC106S catalytic mutant were calculated using the Mann–Whitney test (**P < 0.01, ***P < 0.001).

A3A enzymes are orthologous across placental mammals (33). By contrast A3B is a feature of primate genomes at least from Cercopithecus monkeys to man. Phylogenic analysis of A3A/A3Bc pairs from primates, revealed that rhesus macaque rhA3A and rhA3Bc clustered together and apart from the A3A or A3Bc groups defined by the human, chimp and bonobo enzymes suggesting some recent gene conversion (Supplementary Figure S4A). Of the 22 residues (11%) distinguishing rhA3A and rhA3Bc only six sites were also variable between human A3A and A3Bc (Figure 4A). To see if the same genotoxic dichotomy existed for the rhesus enzymes, four rhA3A and rhA3B constructs were made, found to be well expressed and were localized comparably to their human counterparts (Figure 4B and C). RhA3B and rhA3Bc were unable to produce DSBs unlike rhA3A and rhA3AP2, so paralleling their human counterparts (Figure 4D, Supplementary Figure S3D). Of the macaque A3A/B residues corresponding to human HT16DT and K213Q only residue 16 was variable and then as a known D/N polymorphism (Figure 4A, boxed) (33). Overall the substitutions in both human and macaque A3Bc domains make them more acidic compared to A3A that could reduce the overall affinity for negatively charged ssDNA. Interestingly, this observation extends to every available A3Bc sequence, being consistently the most acidic of all A3 domains (Supplementary Figure S4B).

In view of the attenuated yet conserved A3B phenotype compared to A3A, we wondered if it could contribute to human variation by looking at the RNAseq transcriptome in three purified mature sperm samples (Supplementary Figure S5). *APOBEC* transcript frequencies were of the order of 4–16 × 10^−5^ of total reads compared to the sperm specific protamine *PRM1* gene which was correctly expressed. No *A3B* transcripts were identified. *APOBEC4* encodes a protein with clear identity to other APOBEC enzymes yet is devoid of catalytic activity. *APOBEC4* levels were a little higher (3–9 × 10^−4^ of total reads) but still very low compared to *PRM1* that contrasts with abundant *APOBEC4* expression in human testis (34). As ∼60% of sperm show motility defects, the very small number of *APOBEC* transcripts, which were not consistent between samples, most likely means that they are without consequence in sperm.

## DISCUSSION

While it is now appreciated that A3A and A3B are both endogenous human DNA mutators, their phenotypes are different and non-overlapping. A3A expression is tightly regulated, with steady state levels of A3A kept very low in most cells. Despite the small fraction of A3A localizing to the nucleus, A3A is the more genotoxic of the two (Figures 1F and 2C). In addition, A3A expression is very sensitive to IFNα, IFNγ and PMA (11,25,35,36). By contrast A3B is strictly nuclear, expressed in most tissues and is insensitive to INFα. However, if both enzymes are pro-apoptotic and can introduce mutations in nuDNA as detected by highly sensitive 3DPCR (6,9,25), A3B consistently failed to generate DSBs above background levels (6). The underlying mechanisms for this dichotomy are several. First, the N-terminal A3Bn domain plays a major role in attenuating A3B function for the introduction of clusters 1, 2 and 3 residues from A3A fail to rescue a DSB formation phenotype as they do in a A3Bc backbone (Figure 2C). In addition when fused to the N-terminus of A3A, it attenuated A3A function (Figure 2C). The molecular basis underlying the negative effect of A3Bn could include specific protein interactors as well as A3B dimerization.

Second, exchange of clusters 1 and 2 residues from A3A clearly conferred DSB capacity on the chimeric A3Bc constructs (Figure 2C). Of the eight substitutions and the deletion present in cluster 1, D196H, T197H and Q213K appeared crucial for DSB formation, the latter being the most important (Figure 3B). Nonetheless, the quadruple A3Bc mutant DT196HI+RQ212HK only recovered ∼50% of DSB activity compared to A3A (Figure 3A) suggesting that other cluster 1 substitutions are involved whose individual contributions do not score above background. The effects of residues defining cluster 2 are equally subtle and context dependent, since replacement of three residues alone in A3Bc had no effect on DSB induction (Figure 3A). Clusters 2 and 3 mutations together (A3Bc2–3) performed as well as cluster 1 mutations alone (A3Bc1) and A3Ap1 and A3Ap2 indicating a certain degree of overlap and degeneracy.

Interestingly, the genotoxic dichotomy between A3A and A3B was also apparent for the rhesus macaque enzymes. Although the 22 residues distinguishing the catalytic domains of two enzymes were more widely distributed across the sequence (Figure 4A). Of the three residues distinguishing human A3A and A3B only D16N was variable. However, as this is polymorphic between equally functional rhA3A enzymes (33) it cannot explain the difference. As the rhesus A3A and A3B sequences cluster together and not with the human, chimp and bonobo clusters of A3A or A3B sequences perhaps a more recent gene conversion event other residues underwent selection to maintain the A3A/A3B genotoxic dichotomy.

*A3B* deletion haplotype frequencies of 30–40% in SE Asia (37) not only reinforce the observation that the A3B phenotype is weak but also that is not required for survival and fertility. The RNAseq analysis of purified mature human sperm shows that endogenous human cytidine deaminases may be principally active in the soma and may not be a source of male heritable diversity. While recently evolved human genomes show a penchant for 5′TCC → 5′TTC transitions their frequency was the same for Chinese and Yoruba populations which differ considerably in terms of the *A3B* deletion allele (37) again arguing against A3A and A3B playing a role in evolution of the human genome (38). Nonetheless, the conservation of attenuated A3B genotoxicity compared to A3A across primates argues for an essential role in somatic cells, albeit a subtle one.

## Supplementary Material

SUPPLEMENTARY DATA
