# Supplementary material for: Molecular basis of the attenuated phenotype of human APOBEC3B DNA mutator enzyme
Source: Nucleic Acids Res. 2015 Sep 17;43(19):9340–9. doi: 10.1093/nar/gkv935 (PMC4627089; doi:10.1093/nar/gkv935)
Supplement: SUPPLEMENTARY DATA [file supp_43_19_9340__index.html]

Molecular basis of the attenuated phenotype of human APOBEC3B DNA mutator enzyme — SUPPLEMENTARY DATA 

# Molecular basis of the attenuated phenotype of human APOBEC3B DNA mutator enzyme

## SUPPLEMENTARY DATA

- SUPPLEMENTARY DATA
- SUPPLEMENTARY DATA
