# Supplementary material for: Molecular basis of the attenuated phenotype of human APOBEC3B DNA mutator enzyme
Source: Nucleic Acids Res. 2015 Sep 17;43(19):9340–9. doi: 10.1093/nar/gkv935 (PMC4627089; doi:10.1093/nar/gkv935)
Supplement: SUPPLEMENTARY DATA [file supp_gkv935_nar-01816-d-2015-File006.pdf]

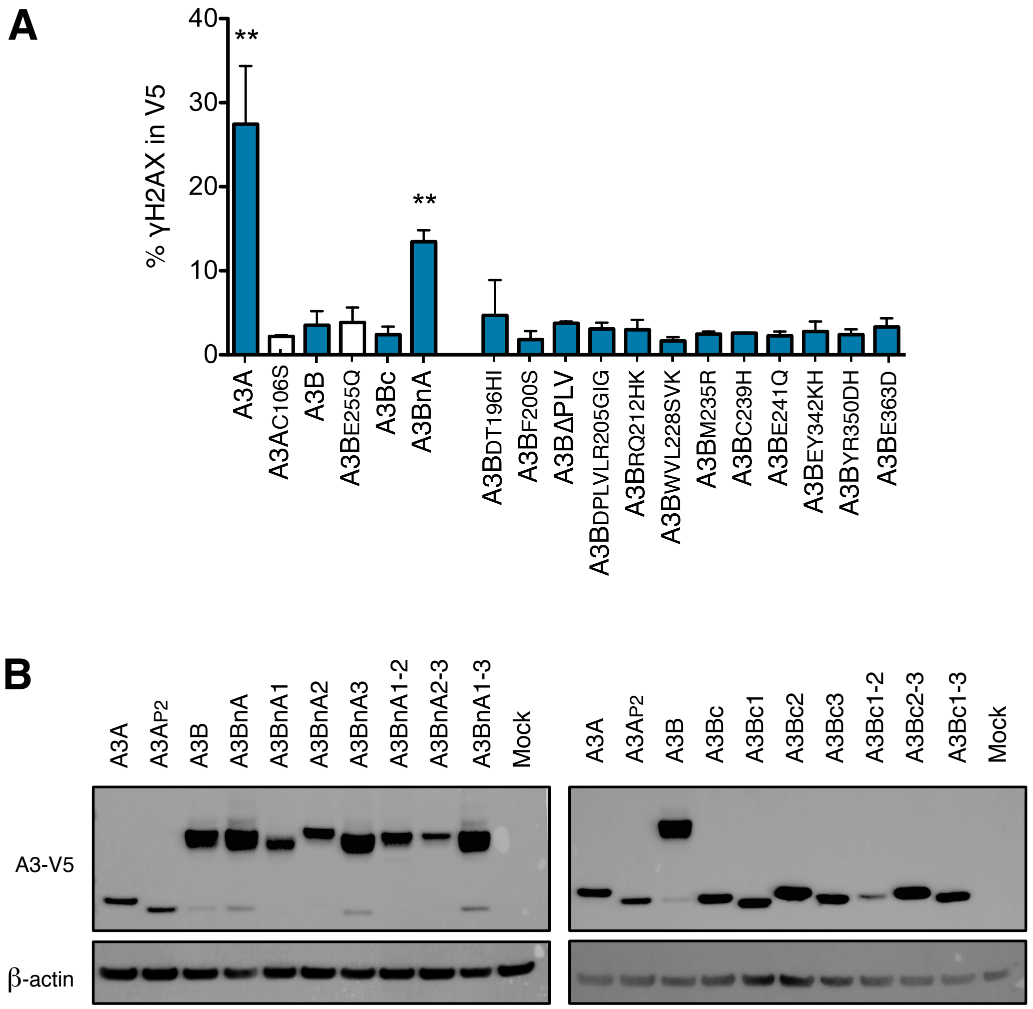

Supplementary Figure S1 : Fluorescence-activated cell sorting analysis of gH2AX-positive cells and western blot analysis of V5-tagged A3 chimeras.

A) Fluorescence-activated cell sorting (FACS) analysis of γH2AX-positive HeLa cells gated on V5-positive cells after A3B mutants transfection at 48 h. Error bars represent s.d. from four independent transfections. Differences compared with A3AC106S catalytic mutant were calculated using the Mann–Whitney test (** $p<0.01$). B) Western blot analysis of V5-tagged A3 chimeras in human HEK 293T cells. β-Actin was used as loading control.

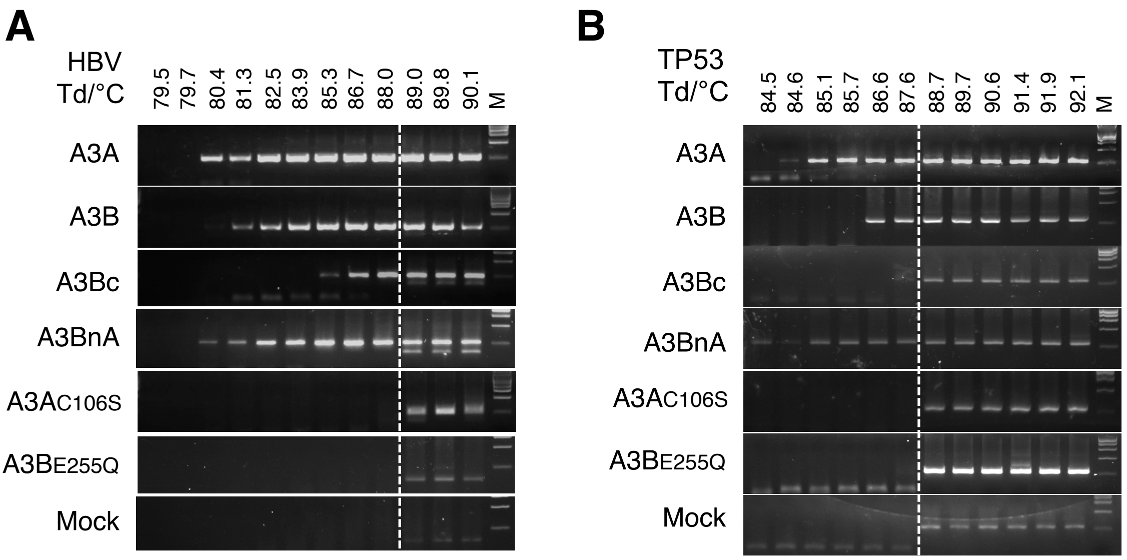

Supplementary Figure S2. A3 editing on HBV DNA and nuclear DNA.
A) HBV genome-specific 3DPCR gels after QT6 cells transfections with A3 coding plasmids.
B) TP53 specific 3DPCR gels after 293T-UGI cells transfections with A3 coding plasmids.

A

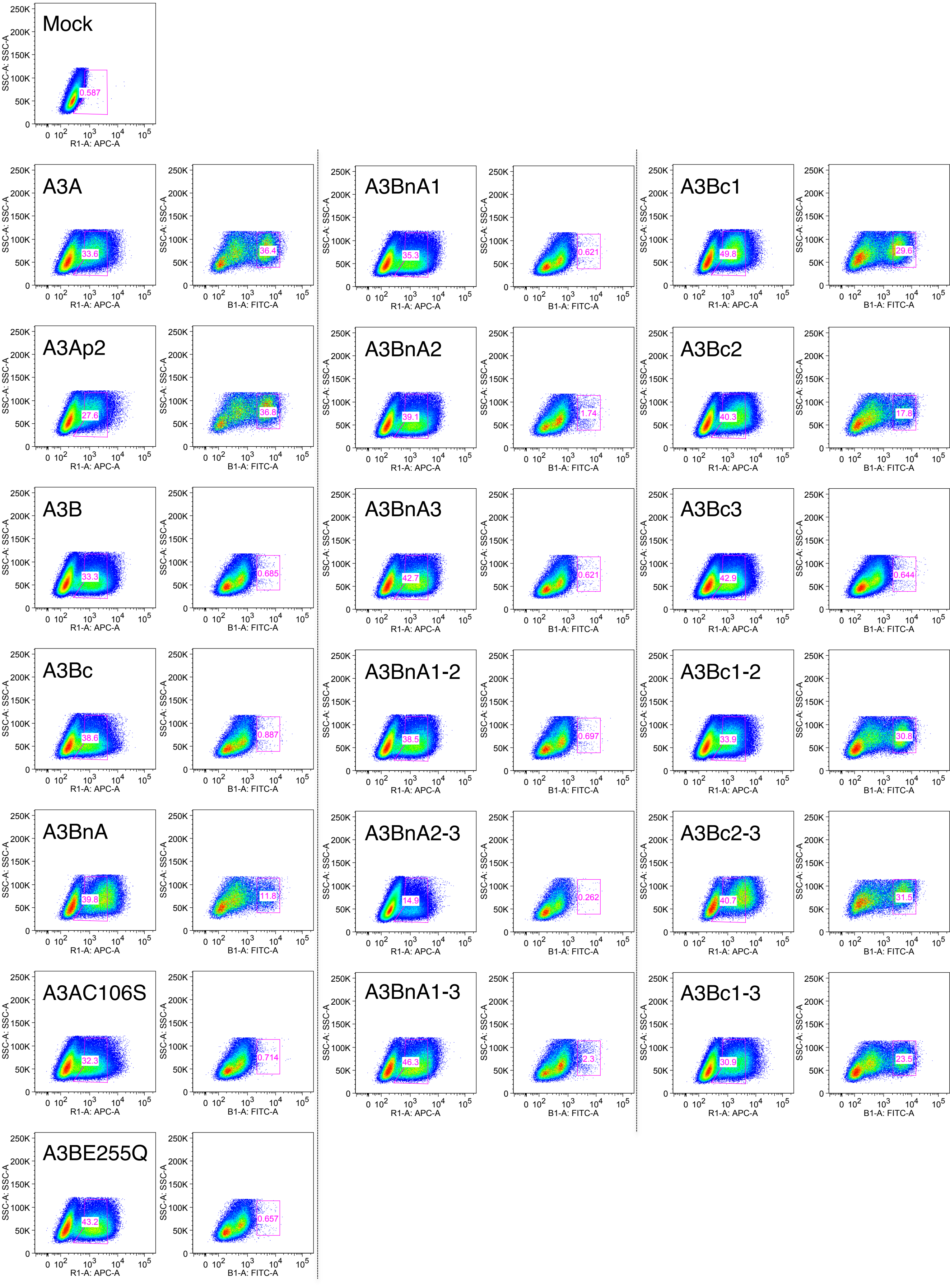
Mock
0.587
A3A
33.6
36.4
A3Ap2
27.6
36.8
A3B
33.3
0.685
A3Bc
38.6
0.887
A3BnA
39.8
11.8
A3AC106S
32.3
0.714
A3BE255Q
43.2
0.657
A3BnA1
35.3
0.621
A3BnA2
39.1
1.74
A3BnA3
42.7
0.621
A3BnA1-2
38.5
0.697
A3BnA2-3
14.9
0.262
A3BnA1-3
46.3
2.3
A3Bc1
49.8
29.6
A3Bc2
40.3
17.8
A3Bc3
42.9
0.644
A3Bc1-2
33.9
30.8
A3Bc2-3
40.7
31.5
A3Bc1-3
30.9
23.5
SSC-A: SSC-A
R1-A: APC-A
B1-A: FITC-A

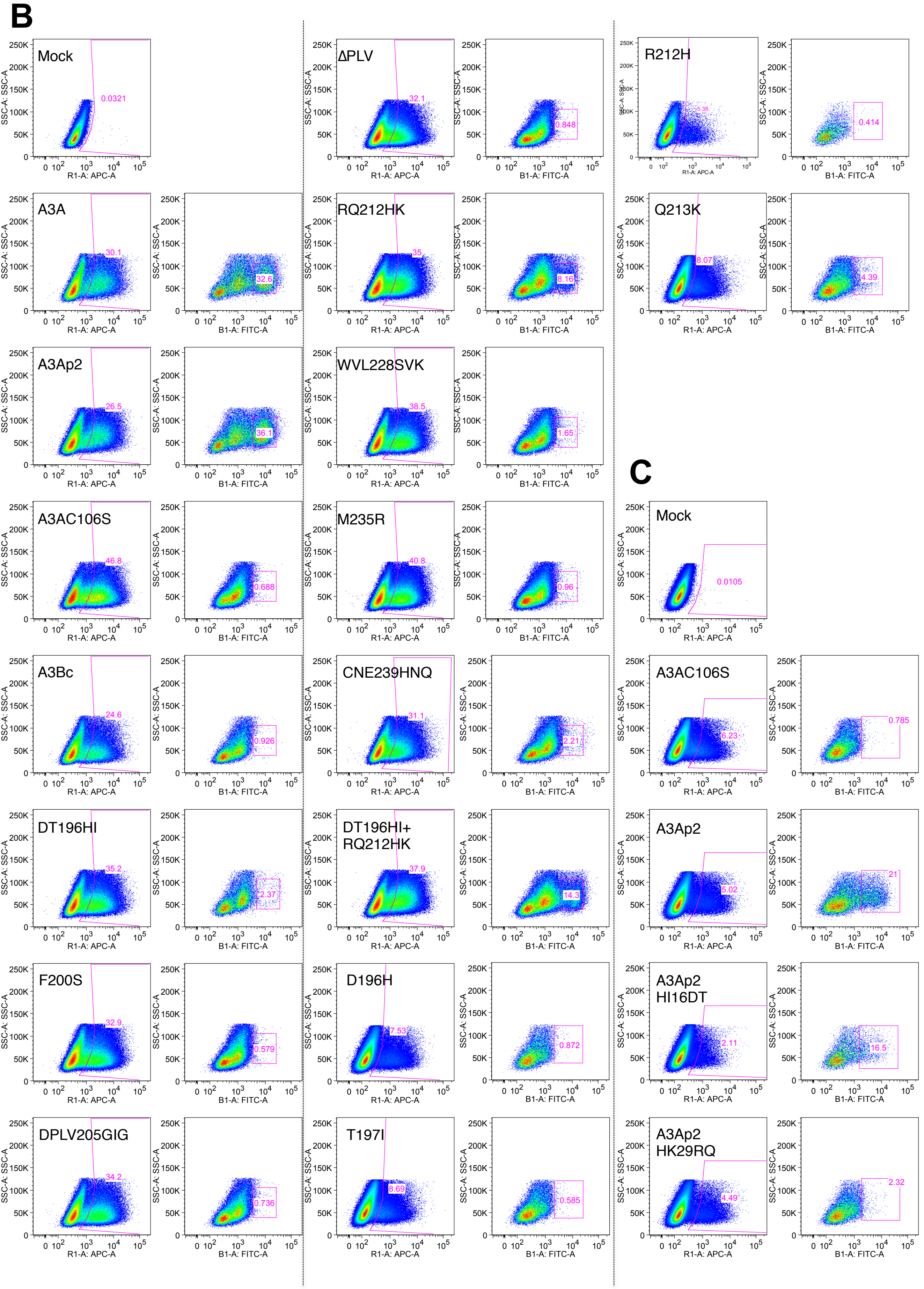
B
Mock
0.0321
ΔPLV
32.1
0.848
R212H
5.38
0.414
A3A
30.1
32.6
RQ212HK
35
8.16
Q213K
8.07
4.39
A3Ap2
26.5
36.1
WVL228SVK
38.5
1.65
A3AC106S
46.8
0.688
M235R
40.8
0.96
A3Bc
24.6
0.926
CNE239HNQ
31.1
2.21
DT196HI
35.2
2.37
DT196HI+
RQ212HK
37.9
14.3
F200S
32.9
0.579
D196H
7.53
0.872
DPLV205GIG
34.2
0.736
T197I
8.69
0.585
C
Mock
0.0105
A3AC106S
6.23
0.785
A3Ap2
5.02
21
A3Ap2
HI16DT
2.11
16.5
A3Ap2
HK29RQ
4.49
2.32
SSC-A: SSC-A
R1-A: APC-A
B1-A: FITC-A
250K
200K
150K
100K
50K
0
0 10^2 10^3 10^4 10^5

D

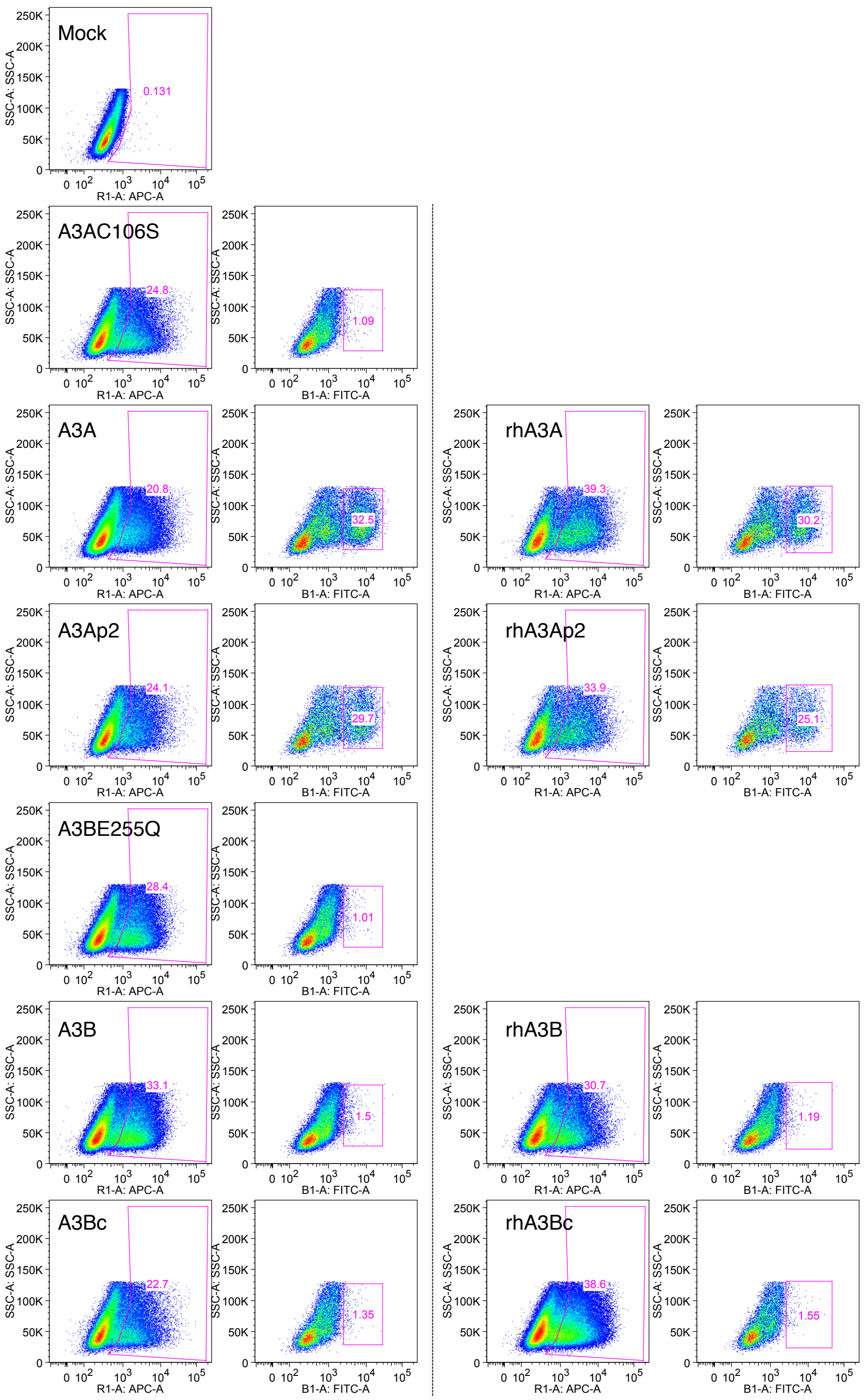

Supplementary Figure S3 : Fluorescence-activated cell sorting analysis of Double strand breaks formation after A3 transfection.

FACS pseudo-color plots of γH2AX staining (FITC window, right panel) performed on V5 positive HeLa cells (APC window, left panel) after A3 (2 µg) transfection at 48 hours. A3AC106S catalytic mutant transfection is used as negative control.

A

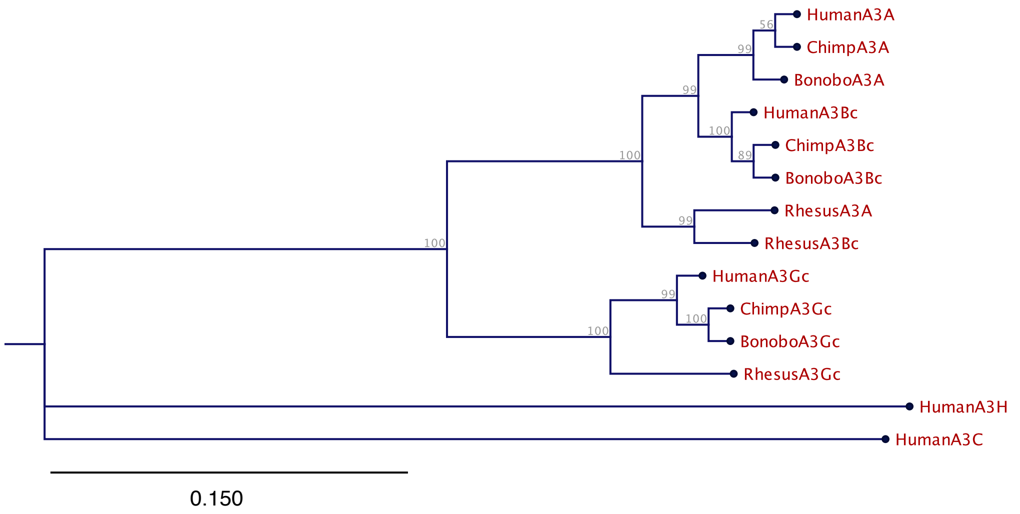

B

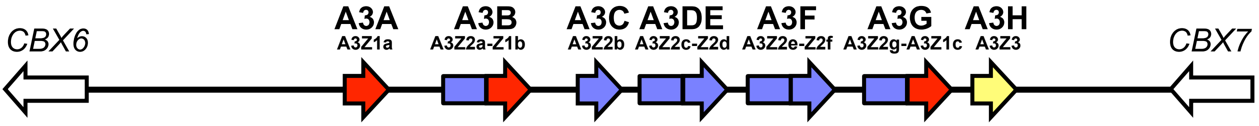

	A3AP2	A3Bc	A3Gc	A3Bn	A3DEn	A3Fn	A3Gn	A3DEc	A3Fc	A3C	A3H
Human	6.5	4.9	5.9	7.8	9.0	8.3	9.4	7.4	5.7	7.4	8.6
Chimp	6.9	5.0	6.6	7.1	9.5	8.6	9.3	7.2	5.5	7.9	8.4
Bonobo	6.6	5.0	6.5	6.7	9.5	-	9.5	6.6	-	7.7	8.4
Gibbon	6.7	5.2	7.3	6.7	9.5	-	9.5	7.1	-	8.2	9.6
Rhesus	7.1	4.6	6.1	7.8	8.6	8.6	9.3	6.0	6.2	7.5	9.9
Mean ±S.D	6.8 ±0.2	4.9 ±0.2	6.5 ±0.5	7.2 ±0.6	9.2 ±0.4	8.5 ±0.2	9.4 ±0.1	6.9 ±0.6	5.8 ±0.4	7.7 ±0.3	9.0 ±0.7

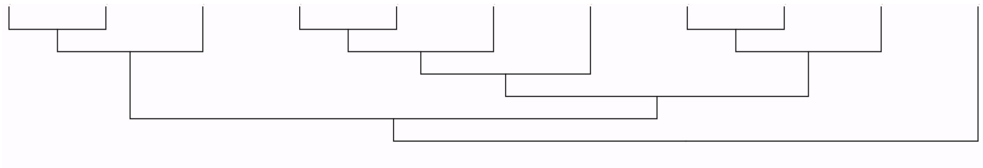

Supplementary Figure S4.

Evolutionary history of A3A and A3B Z1 domain and theoretical Isoelectric point on primate A3 domains

A) Numbers correspond to bootstrap values inferred from 1,000 replicates. Evolutionary distances were computed using Poisson correction method and are expressed in numbers of substitution per site.

B) Theoretical isoelectric point (pI) performed on primate A3 domains using primary amino acid sequences. Values were rounded-off to one digit after the decimal. A3 domains are organized following human A3 domains phylogeny.

Gene ID	Sample 1	Sample 2	Sample 3
AID	0	0	1
APOBEC1	0	0	1
APOBEC2	1	2	2
APOBEC3A	0	6	0
APOBEC3B	0	0	0
APOBEC3C	0	8	1
APOBEC3DE	0	6	0
APOBEC3F	0	8	2
APOBEC3G	0	11	2
APOBEC3H	0	5	0
APOBEC4	18	19	24
PRM1	26539	69783	27855
HPRT1	0	0	0

Supplementary Figure S5.

Human polynucleotide cytidine deaminase transcriptome in purified mature human sperm. Absolute numbers of RNAseq transcripts identified. *PRM1* is the sperm specific protamine 1 gene while *HPRT1* is known not to be expressed in sperm.
