# Supplementary material for: Molecular basis of the attenuated phenotype of human APOBEC3B DNA mutator enzyme
Source: Nucleic Acids Res. 2015 Sep 17;43(19):9340–9. doi: 10.1093/nar/gkv935 (PMC4627089; doi:10.1093/nar/gkv935)
Supplement: SUPPLEMENTARY DATA [file supp_gkv935_nar-01816-d-2015-File007.pdf]

Plasmid	Matrix	Primers
A3Bc	A3Bi7	For : 5′-CACC**ATG**GATCCAGACACATTCACTTTCAACTTTA-3′<br>Rev : 5′-GTTTCCCTGATTCTGGAGAAT-3′
A3BnA	Puc57-A3BnA	For : 5′-CACC**ATG**AATCCACAGATCAGAAATCCGATGGAGC-3′<br>Rev : 5′-GTTTCCCTGATTCTGGAAGAGCAGGGGGGTTG-3′
A3BnA1	A3BnA	For : 5'-CACC**ATG**AATCCACAGATCAGAAATCCG-3'<br>Rev : 5'-GCCATTGTCCAGGCGCTCCACTTCGTAGCACAG-3'
	A3Bi7	For : 5'-CTGTGCTACGAAGTGGAGCGCCTGGACAATGGC-3'<br>Rev : 5'-GTTTCCCTGATTCTGGAAGAGCAGGGGGGTTG-3'
A3BnA2	A3BnA	For : 5'-CACC**ATG**AATCCACAGATCAGAAATCCG-3'<br>Rev : 5'-GCCATTGTCCAGGCGCTCCACTTCGTAGCACAG-3'
	A3Bi7	For : 5'- CTGTGCTACGAAGTGGAGCGCCTGGACAATGGC-3'<br>Rev : 5'- TATAAGGAGGCGCTGCAAATGCTGCGGGATGCT-3'
A3BnA3	A3Bi7	For : 5'-CACC**ATG**AATCCACAGATCAGAAATCCG-3'<br>Rev : 5'-AGCATCCCGCAGCATTTGCAGCGCCTCCTTATA-3'
	A3BnA	For : 5'-TATAAGGAGGCGCTGCAAATGCTGCGGGATGCT-3'<br>Rev : 5'-GTTTCCCTGATTCTGGAAGAGCAGGGGGGTTG-3'
A3BnA1-2	A3BnA	For : 5'-CACC**ATG**AATCCACAGATCAGAAATCCG-3'<br>Rev : 5'-AGCATCCCGCAGCATTTGCAGCGCCTCCTTATA-3'
	A3Bi7	For : 5'-TATAAGGAGGCGCTGCAAATGCTGCGGGATGCT-3'<br>Rev : 5'-GTTTCCCTGATTCTGGAAGAGCAGGGGGGTTG-3'
A3BnA1-3	A3BnA	For : 5'-CACC**ATG**AATCCACAGATCAGAAATCCG-3'<br>Rev : 5'-AGCATCCCGCAGCATTTGCAGCGCCTCCTTATA-3'
	A3Bi7	For : 5'-TATAAGGAGGCGCTGCAAATGCTGCGGGATGCT-3'<br>Rev : 5'-GTTTCCCTGATTCTGGAAGAGCAGGGGGGTTG-3'
A3BnA2-3	A3Bi7	For : 5'-CACC**ATG**AATCCACAGATCAGAAATCCG-3'<br>Rev : 5'-GCCATTGTCCAGGCGCTCCACTTCGTAGCACAG-3'
	A3BnA	For : 5'-CTGTGCTACGAAGTGGAGCGCCTGGACAATGGC-3'<br>Rev : 5'-GTTTCCCTGATTCTGGAAGAGCAGGGGGGTTG-3'
A3Bc1<br>A3Bc1-2<br>A3Bc1-3	A3BnA1<br>A3BnA1-2<br>A3BnA1-3	For : 5'-CACC**ATG**GATCCACACATATTCACTTCC-3'<br>Rev : 5'-GTTTCCCTGATTCTGGAAGAGCAGGGGGGTTG-3'
A3Bc2<br>A3Bc2-3<br>A3Bc3	A3BnA2<br>A3BnA2-3<br>A3BnA3	For : 5'-CACC**ATG**GATCCACACATATTCACTTCC-3'<br>Rev : 5'-GTTTCCCTGATTCTGGAAGAGCAGGGGGGTTG-3'
Rhesus-A3B	Puc57-RhesusA3B	For : 5'-CACC**ATG**AATCCACAGATCAGAAATCCG-3'<br>Rev : 5'-GTTTCCCTGATTCTGGAGAATGGC-3'
Rhesus-A3Bc	Puc57-RhesusA3B	For : 5'-CACC**ATG**GATCCAGACACGTTCACTTCCAA-3'<br>Rev : 5'-GTTTCCCTGATTCTGGAAGAGCAGGGGGGTTG-3'
Rhesus-A3Ap2	Rhesus-A3A	For : 5'-CACC**ATG**GATCCAAACACGTTCACTTTCAAC-3'<br>Rev : 5'-GTTTCCCTGATTCTGGAGAATGGC-3'
A3Ap2HI16DT	A3A	For : 5'-CACC**ATG**GATCCA**GACACA**TTCACTT-3'<br>Rev : 5'-GTTTCCCTGATTCTGGAGAATGGC-3'
A3BcD96H	A3Bc	For : 5'-CACC**ATG**GATCCA**CAC**ACATTCACTT-3'<br>Rev : 5′-GTTTCCCTGATTCTGGAGAAT-3′
A3BcT97I	A3Bc	For : 5'-CACC**ATG**GATCCAGAC**ATA**TTCACTT-3'<br>Rev : 5′-GTTTCCCTGATTCTGGAGAAT-3′

Table T1. Compendium of primers used to generate A3A/A3B chimeras coding sequences for pcDNA3.1 TOPO TA V5 cloning.

**Plasmid**	**Matrix**	**Primers**
A3BDT196HI	A3Bi7	For : 5'-TACCTGATGGATCCA**CACATA**TTCACTTTCAACTTT-3'<br>Rev : 5'-AAAGTTGAAAGTGAA**TATGTG**TGGATCCATCAGGTA-3'
A3BF200S	A3Bi7	For : 5'-CCAGACACATTCACT**TCC**AACTTTAATAATGAC-3'<br>Rev : 5'-GTCATTATTAAAGTT**GGA**AGTGAATGTGTCTGG-3'
A3BΔPLV	A3Bi7	For : 5′-TTCAACTTTAATAATGACCTTCGACGGCGCCAGACC-3′<br>Rev : 5′-GGTCTGGCGCCGTCGAAGGTCATTATTAAAGTTGAA-3′
A3BDPLVLR205GIG	A3Bi7	For : 5'-TTCAACTTTAATAAT**GGCATTGGA**CGGCGCCAGACCTAC-3'<br>Rev : 5'-GTAGGTCTGGCGCCG**TCCAATGCC**ATTATTAAAGTTGAA-3'
A3BRQ212HK	A3Bi7<br>A3BDT196HI	For : 5'-TTGGTCCTTCGACGG**CATAAG**ACCTACTTGTGCTAT-3'<br>Rev : 5'-ATAGCACAAGTAGGT**CTTATG**CCGTCGAAGGACCAA-3'
A3BWVL228SVK	A3Bi7	For : 5'-CTGGACAATGGCACC**TCGGTCAAG**ATGGACCAGCACATG-3'<br>Rev : 5'-CATGTGCTGGTCCAT**CTTGACCGA**GGTGCCATTGTCCAG-3'
A3BM235R	A3Bi7	For : 5'-CTGATGGACCAGCAC**AGG**GGCTTTCTATGCAAC-3'<br>Rev : 5'-GTTGCATAGAAAGCC**CCT**GTGCTGGTCCATCAG-3'
A3BC239H	A3Bi7	For : 5'-CACATGGGCTTTCTA**CAC**AACGAGGCTAAGAAT-3'<br>Rev : 5'-ATTCTTAGCCTCGTT**GTG**TAGAAAGCCCATGTG-3'
A3BE241Q	A3Bi7	For : 5'-GGCTTTCTATGCAAC**CAG**GCTAAGAATCTTCTCTGT-3'<br>Rev : 5'-ACAGAGAAGATTCTT**AGC**CTGGTTGCATAGAAAGCC-3'
A3BCNE239HNQ	A3Bi7	For : 5'-CACATGGGCTTTCTA**CAC**AAC**CAG**GCTAAGAATCTTCTCTGT-3'<br>Rev : 5'-ACAGAGAAGATTCTT**AGC**CTG**GTT**GTGTAGAAAGCCCATGTG-3'
A3BEY342KH	A3Bi7	For : 5'-ACCTACGATGAGTTT**AAGCAC**TGCTGGGACACCTTT-3'<br>Rev : 5'-AAAGGTGTCCCAGCA**GTGCTT**AAACTCATCGTAGGT-3'
A3BYR350DH	A3Bi7	For : 5'-TGGGACACCTTTGTG**GACCAC**CAGGGATGTCCCTTC-3'<br>Rev : 5'-GAAGGGACATCCCTG**GTGGTC**CACAAAGGTGTCCCA-3'
A3BE363D	A3Bi7	For : 5'-CCCTGGGATGGACTA**GAT**GAGCACAGCCAAGCC-3'<br>Rev : 5'-GGCTTGGCTGTGCTC**ATC**TAGTCCATCCCAGGG-3'
A3BcR212H	A3Bc	For : 5'-TTGGTCCTTCGACGG**CATCAG**ACCTACTTGTGCTAT-3'<br>Rev : 5'-ATAGCACAAGTAGGT**CTGATG**CCGTCGAAGGACCAA-3
A3BcQ213K	A3Bc	For : 5'-TTGGTCCTTCGACGG**CGCAAG**ACCTACTTGTGCTAT-3'<br>Rev : 5'-ATAGCACAAGTAGGT**CTTGCG**CCGTCGAAGGACCAA-3'

Table T2. Compendium of primers used for site directed mutagenesis on A3B expression plasmids.
